# Proteomic signature of aging in bloodstain samples: a preliminary study

**DOI:** 10.1186/s12864-025-12164-x

**Published:** 2025-10-29

**Authors:** Niu Gao, Daijing Yu, Jingjing Xu, Jiaxuan Hao, Jinding Liu, Jiangwei Yan

**Affiliations:** 1 School of Forensic Medicine, Shanxi Medical University, No. 55 Wenhua Street, Yuci District, Jinzhong, 030600 Shanxi China; 2 Shanxi Key Laboratory of Forensic Medicine, No. 55 Wenhua Street, Yuci District, Jinzhong, 030600 Shanxi China

**Keywords:** Age prediction, Bloodstain, Orbitrap astral, Proteomics

## Abstract

**Background:**

Age estimation from biological samples remains a critical challenge in forensic investigations, particularly when analyzing trace or degraded biological stains recovered from crime scenes. While DNA methylation has gained attention as a potential epigenetic clock for age prediction, practical limitations that include the requirements for bisulfite conversion and environmental interference hinder its forensic utility. Previous studies have shown that proteins are more stable than DNA and that certain proteins are highly age-related and gender-differentiated, indicating that proteomic signatures present a promising alternative for biological age determination. However, proteomic data from bloodstains have not yet been used for forensic age prediction. In this pilot study, we used the high-resolution Thermo Scientific Orbitrap Astral Mass Spectrometer to investigate the proteomic signatures of aging in bloodstain samples from 40 healthy males (aged 10–79 years) stored for 4 years at room temperature (20–25 °C). Age-related proteins were subsequently selected for age prediction. We further simplified the characteristic variables using the Least Absolute Shrinkage and Selection Operator (Lasso) regression and the Boruta algorithm, and established age prediction models for bloodstains based on Random Forest (RF) machine learning.

**Results:**

In total, 1,655 proteins were identified, which showed four different nonlinear change patterns during the aging process. Pearson’s correlation coefficient (R) was calculated, and 71 proteins were found to correlate significantly with age (Pearson’s |R| > 0.3, *P* < 0.05), including 26 positively and 45 negatively correlated proteins. Functional enrichment analysis revealed that age-associated proteins were markedly enriched in pathways related to endocytosis, metabolism, and neurodegenerative disease. Feature selection using the Lasso regression and the Boruta algorithm resulted in the identification of 18 and 10 age-associated proteins, respectively, with six overlapping proteins (including *ITIH3*, *HSPA9*, *SNAP91*, *FTL*, *XPO4*, and *NCF2*). RF regression models were constructed using different feature sets: Lasso-selected (18 proteins), Boruta-selected (10 proteins), their intersection (6 proteins), and R-value-based filtering (e.g., top 7 proteins with |R| > 0.4, *P* < 0.05). The Boruta-based model demonstrated the highest predictive accuracy, achieving an R² of 0.70 and a Mean Absolute Error (MAE) of 9.14 years for the testing set.

**Conclusions:**

These findings demonstrate the potential of bloodstain proteomics for age estimation. This study provides a foundation for further validation in larger cohorts and diverse forensic scenarios.

**Supplementary Information:**

The online version contains supplementary material available at 10.1186/s12864-025-12164-x.

## Introduction

In forensic science, precise estimation of the age of biological evidence at the crime scene is a key technical component of any investigation. By determining the chronological age range of the suspect, the scope of the investigation can be effectively narrowed, which is valuable for improving the efficiency of investigations. With the development of age-related research, molecular biomarkers such as amino acid racemization [1], T cell DNA rearrangements [2], telomere length [3], advanced glycation end products [3], RNA molecules [4], and DNA methylation [5] are increasingly being used for age prediction. Among these molecular markers, DNA methylation has been considered the most promising biomarker owing to its high prediction accuracy [5]; however, bisulfite conversion of DNA may lead to DNA breakage or degradation [6]. In addition, a variety of factors such as diet, stress, and smoking behavior also affect the process of methylation [7]. Hence, this method is unsatisfactory for the detection of forensic trace samples, limiting its application in forensic practice. Accurate age prediction methods suitable for trace and degraded samples are still lacking, and new molecular markers for age prediction are required.

The rapid development of proteomics technology in the past decade has provided new perspectives for forensic age prediction. Aging, as a complex biological process, is characterized by a progressive loss of physiological integrity, leading to impaired function and increased risk of death. This process involves loss of proteostasis [8], indicating that proteins may be used as biomarkers for age estimation. Proteomics, which complements genomics and transcriptomics [9], is considered the most relevant single dataset for characterizing biological systems [10]. Therefore, proteome profiling (including proteins and their abundance) provides a better global picture of a sample than static DNA information [11]. Furthermore, previous studies have shown that proteins are considerably more stable than DNA [12], indicating that when DNA is unavailable or degraded, proteins may still be present and can be analyzed. These properties of the proteome provide unique advantages in forensic studies.

Fortunately, various studies have reported that the expression of certain proteins is highly age-related. Recent advancements in blood proteomics have revealed age-dependent fluctuations in the expression of more than one-third of plasma proteins [13]. Based on SomaScan aptamer technology, Lehallier et al. and Tanaka et al. identified an independent plasma proteome clock consisting of 373 proteins [13] and 76 proteins [14], respectively, with very high accuracy in predicting age (*r* = 0.93–0.97). *GDF15* is one of the most important age-related proteins, and its expression level increased significantly with age [14]. Johnson et al. systematically evaluated 36 different human proteomes from 32 different aging studies spanning a diverse set of tissues, including blood (serum and plasma), urine, and saliva, and showed that 32 proteins identified in ≥ 5 analyses were all associated with aging and/or age-related diseases [15]. Furthermore, the authors constructed an aging clock based on 83 plasma proteins reported to be age-related in ≥ 3 studies, which had a Mean Absolute Error (MAE) of 5.5 years for predicting actual age in the validation set [15]. These findings provide a solid theoretical basis for inferring forensic age based on proteomics.

As the most common biological material in crime scenes, bloodstains contain proteomic information with unique application potential. Although age-dependent alterations in plasma or serum protein levels have been reported, these findings cannot be directly applied to dried bloodstains, as bloodstain storage conditions affect proteome stability [16]. Fortunately, the proteome of bloodstains has been studied extensively. Bjorkesten et al. detected 92 proteins in dried blood spots using multiplex proximity extension assays and proposed that the drying process only slightly affected the detection of blood proteins [17]. Chambers et al. developed liquid chromatography multiple reaction monitoring mass spectrometry to accurately quantify 97 proteins in dried blood spots [18]; subsequently, Eshghi et al. extended the protein multiplexing capability to 200 proteins [19]. These studies have demonstrated the presence of multiple proteins in bloodstains. However, studies utilizing proteomic data from bloodstains for forensic age prediction are lacking.

The Thermo Scientific Orbitrap Astral Mass Spectrometer (MS) combines a mass-resolving quadrupole, the Orbitrap, and the novel Asymmetric Track Lossless (Astral) analyzer to further extend the achievable sensitivity and acquisition speed [20]. Combined with data-independent acquisition (DIA), the analyzer quantifies up to five times more peptides per unit time than the Thermo Scientific Orbitrap Mass Spectrometer, with powerful features such as high acquisition rate, high resolution, high sensitivity, and automated gain control, providing a previously unattainable dynamic range for proteomic testing [21]. These advancements in mass spectrometry technology would likely lead to improved resolution/coverage of protein signals in aged bloodstain samples.

In this pilot study, we aimed to investigate the proteomic signature of aging in bloodstain samples using the Thermo Scientific Orbitrap Astral Mass Spectrometer and identify age-related proteins. We then used Boruta and Least Absolute Shrinkage and Selection Operator (Lasso) feature screening methods to select proteins and used Random Forest (RF) to build age prediction models.

## Materials and methods

### Study population

To eliminate the confounding factor of gender in proteomic aging studies, fingerstick blood was collected from 40 healthy male individuals (aged 10–79 years). Finger pricks were performed by pre-service trained and certified blood collectors using sterile disposable lancets. The first drop of blood from the finger-prick was wiped off using a dry sterile cotton ball. Gentle and intermittent pressure was then applied to the tissue around the puncture point to increase blood flow. The subsequent blood drops were collected onto a Flinders Technology Associates (FTA) card. Blood spots were then dried at room temperature (20–25 °C) for 2 h, with minimal exposure to intense light. Finally, the samples were placed in ziplock bags containing desiccant and stored at room temperature for four years to prepare aged bloodstain samples. The study was approved by the Institutional Review Board of Shanxi Medical University (Approval Number: No. 2020GLL031) and conformed to the guidelines of the World Medical Association and the Declaration of Helsinki. All donors volunteered for this study and provided informed consent. For participants under the age of sixteen, informed consent was provided by their parents or legal guardians. All collection methods complied with the relevant guidelines and regulations.

### Bloodstain proteomic profiling workflow

#### Protein extraction and digestion

Two holes (diameter = 5.0 mm) were punched from the bloodstain spot and cut into eight smaller pieces, which were placed in a 1.5 mL polypropylene tube. The bloodstain samples were lysed by adding 500 µL of DB protein lysate [8 M urea, 100 mM triethylammonium bicarbonate (TEAB), pH 8.5] and vortex-mixing for 1 min. The homogenate was centrifuged at 12,000 × *g* for 15 min at 4 °C. The supernatant was collected and reduced using 10 mM dithiothreitol (final concentration) at 56 °C for 1 h and then immediately cooled on ice for 2 min. Subsequently, alkylation was performed by adding iodoacetamide to a final concentration of 45 mM and incubating in the dark at 25 °C for 1 h. The concentration and quality of the extracted proteins were assessed using the Bradford assay and electrophoresis on a 12% sodium dodecyl sulfate polyacrylamide gel electrophoresis (SDS-PAGE), respectively.

For digestion, the volume of the bloodstain proteins was made up to 100 µL using the DB protein lysate. The diluted protein was mixed with 1 µg trypsin and 100 mM TEAB buffer and digested at 37 °C for 4 h, followed by overnight digestion using 1 µg trypsin and 0.1% CaCl_2_, where the protein-to-trypsin ratio was 10:1. The pH of the digested proteins was adjusted to less than 3 by adding formic acid (FA) and then mixed and centrifuged at 12,000 × *g* for 5 min at room temperature. The supernatant was slowly passed through a C18 desalting column, and the column was washed three times in succession using a washing buffer [0.1% FA, 3% acetonitrile (ACN)]. The digested proteins were then collected by adding an appropriate volume of eluent (0.1% FA, 70% ACN) and lyophilized.

#### Vanquish Neo UHPLC‑astral LC/MS DIA method

Mobile phase A (0.1% FA) and mobile phase B (0.1% FA and 80% ACN) were prepared. Lyophilized protein powder was dissolved in 10 µL of mobile phase A and centrifuged at 14,000 × *g* for 20 min at 4 °C. The supernatant (200 ng) was injected into a Vanquish Neo upgraded UHPLC system, which had a C18 174,500 pre-column (5 mm × 300 μm, 5 μm, Thermo Fisher Scientific, Waltham, USA) heated at 50 °C in a column warmer. The C18 analytical column used was ES906 (PepMap ^TM^ Neo UHPLC 150 μm × 15 cm, 2 μm, Thermo Fisher Scientific). The liquid chromatography (LC) gradient elution program is described in Table S1.

For proteomic profiling, the Thermo Orbitrap Astral Mass Spectrometer (Thermo Fisher Scientific) was used for high-resolution LC-MS/MS analysis. The separated peptides were subsequently ionized using an easy-spray electrospray ionization source. The ion spray voltage and ion transfer tube temperature were set at 2.0 kV and 290 °C, respectively. The DIA mode was used for the mass spectrum: a single MS1 scan was performed using the Orbitrap analyzer at a resolution of 240,000 (at 200 m/z), with a precursor ion range of m/z 380–980. The automatic gain control, size of the parent ion window, number of DIA windows, and the normalized collision energy were set to 500%, 2-Th, 300, and 25%, respectively. MS2 spectra were acquired using the Astral analyzer with a detection range of 150–2000 m/z, a fragment ion resolution of 80,000, and a maximum injection time of 3 ms. Raw data (.raw) for mass spectrometry detection was obtained.

#### MS data processing

The raw files were searched and analyzed using the DIA-NN software (Cambridge Centre for Proteomics, Cambridge, UK), according to the homo sapiens (human) protein database downloaded from UniProtKB (released on July 26, 2024). The library search parameters were set as follows: the mass tolerance was 10 ppm for precursor ions and 0.02 Da for fragments. An alkylation modification of cysteine was the immobilization modification, and acetylation modification, methionine loss, and methionine loss + acetylation were the N-terminal modifications, allowing for up to two missed cleavage sites. The DIA-NN software further filtered the search results to improve the quality of analysis results: peptide spectrum matches (PSMs) with a ≥ 99% confidence level were considered credible PSMs, and only credible peptides and proteins were retained; false discovery rate (FDR) validation was performed to remove the peptides and proteins with FDR > 1%. Retention time correction was performed using indexed retention time, and the precursor ion Q-value cutoff was set to 0.01. The empirical Bayes method was used to adjust batch effects, and the data were normalized using the median mean center algorithm.

#### Bioinformatics analysis

Functional annotation enrichment analyses were performed for the identified bloodstain proteins. The potential biological functions and protein domains of bloodstain proteins were predicted and analyzed using Gene Ontology (GO) (http://www.geneontology.org/), Kyoto Encyclopedia of Genes and Genomes (https://www.kegg.jp/), and InterPro (IPR; https://www.ebi.ac.uk/interpro/) databases, with the InterProscan software 5.22–61.0 (European Bioinformatics Institute, Hinxton, UK). The GO classifies functions according to three terms: Molecular Function (MF), Biological Process (BP), and Cellular Component (CC). The Clusters of Orthologous Groups of proteins (COGs) and subcellular localization are available at https://www.ncbi.nlm.nih.gov/research/cog and Cell-mPLOC 2.0 (http://www.csbio.sjtu.edu.cn/bioinf/Cell-PLoc-2/), respectively. The construction of clusters of predicted orthologs using seven eukaryotic genomes was named KOGs after eukaryotic orthologous groups.

### Bloodstain protein cluster analysis

To assess the dynamically changing patterns of bloodstain proteins during the whole life span, bloodstain samples were divided into the following four groups according to donor ages: 0–20, 21–40, 41–60, and 61–80 years (*n* = 10 per group). Clustering analysis was performed for the identified proteins in four age groups using the ‘Mfuzz’ package. The algorithm used traditional methods to achieve clustering by calculating membership degrees and cluster centers, where proteins in the same cluster display similar variation trends in expression. Subsequently, GO enrichment analyses (including BP, MF, and CC categories) were performed for proteins in each cluster using the package ‘clusterProfiler’. Cluster analysis was conducted in RStudio software (RStudio Inc., Boston, MA, USA).

### Age-associated proteins and related functions

To identify age-associated proteins, Pearson’s correlation coefficient (R) was calculated, and a linear regression was performed between log10-transformed protein expression levels and chronological age across all individuals. The statistical significance of the regression coefficient was determined using a *t*-test. The cut-off value for R (|R|) was defined as 0.3, and the P value was set to < 0.05. For functional annotation, age-associated proteins were subjected to GO and KEGG enrichment analyses using the ‘clusterProfiler’ package. Enrichment information with P.adjust < 0.05 was considered statistically significant. This analysis aimed to provide insights into the BPs and pathways potentially involved in aging.

### Proteomic prediction of chronological age

To further streamline the important characteristic variables and identify the age-associated proteins, two machine-learning algorithms were used for feature selection. We used the ‘glmnet’ and ‘Boruta’ packages in the RStudio (RStudio Inc., Boston, MA, USA) for feature selection. Lasso regression analysis is a feature selection method that uses the L1-penalty (controlled by lambda) to set the coefficients of less important variables to zero to filter out the significant variables and construct the best classification model. The optimal penalty coefficient λ was determined via Ten-fold cross-validation using the ‘cv.glmnet’ function, selecting the value that minimized the MAE. The Boruta algorithm is built around the RF Classifier algorithm, which identifies the most important features by comparing the Z-score of each feature to the Z-score of the ‘shadow feature’. If the Z-score of a real feature is greater than the maximum Z-score of shaded features, the feature is considered ‘significant’. The Boruta procedure was executed with maxRuns = 100 iterations and doTrace = 2 for progress monitoring.

After feature selection, we constructed the RF prediction model using the Python 3.9 software. The dataset was randomly divided into a training set and a testing set at a 7:3 ratio. The training set was used for model construction and optimization, while the testing set was reserved for evaluating model performance and generalizability to unseen data. Ten-fold cross-validation was applied for internal validation, and hyperparameters were optimized using grid search for each algorithm. The parameter ranges tested were: n_estimators: [200, 300, 500, 700]; max_features: [‘sqrt’, ‘log2’]; max_depth: [1, 3, 5, 10]; min_samples_split: [2, 5, 10]; min_samples_leaf: [1, 2, 4] . The performance of the model was evaluated by obtaining the R^2^, MAE and Root Mean Square Error (RMSE) values between the predicted and chronological ages.

## Results

### Proteomic features of bloodstain proteins

**Fig. 1:**
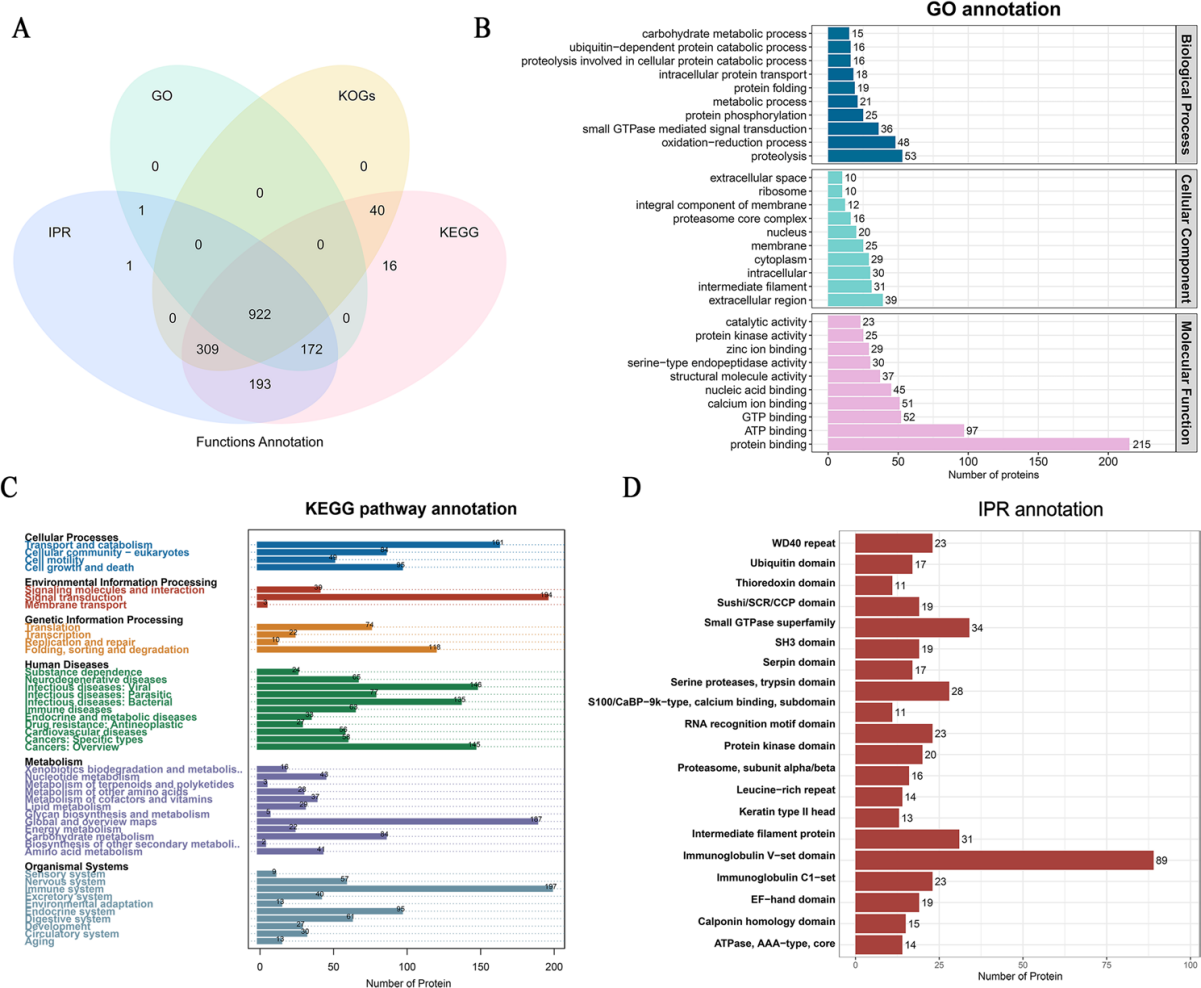
Functional prediction of bloodstain proteins. **A** Venn diagrams show the common and exclusive proteins annotated using the GO, KEGG, IPR, and KOG databases. **B** The top 30 enriched GO terms (including BP, CC, and MF categories). **C** All enriched KEGG terms of level 2 signaling pathways. **D** The top 20 protein domains

In total, 17,675 peptides and 1,655 bloodstain proteins were identified across all 40 samples. The number of bloodstain proteins detected in each sample remained relatively stable, ranging from 1,200 to 1,500 proteins (Fig. S1). To further characterize the properties of bloodstain proteins, the functional annotation of the 1,655 adult bloodstain proteins identified in this study was performed using the GO, KEGG, IPR, and KOG databases (Fig. 1). Of these, 1,654 proteins were successfully annotated, and 922 proteins were simultaneously identified using four databases (Fig. 1A). Among them, 1,095 proteins were enriched in 458 GO items by GO annotation, including 173 BP items, 50 CC items, and 235 MF items, and the top 10 GO terms of each classification are shown in Fig. 1B. In total, 1,652 proteins were annotated using the KEGG database, with the highest number of 197 proteins enriched for the “Immune system” in the item “Organismal systems”. All the KEGG terms of level 2 signaling pathways are shown in Fig. 1C. Furthermore, 1,598 proteins were characterized using IPR functional analysis, including 89 proteins with “Immunoglobulin V-set domain,” and the top 20 protein domains are shown in Fig. 1D. The results of KOGs analysis and subcellular localization are shown in Fig. S2.

### Proteomic features of bloodstain proteins during aging

**Fig. 2:**
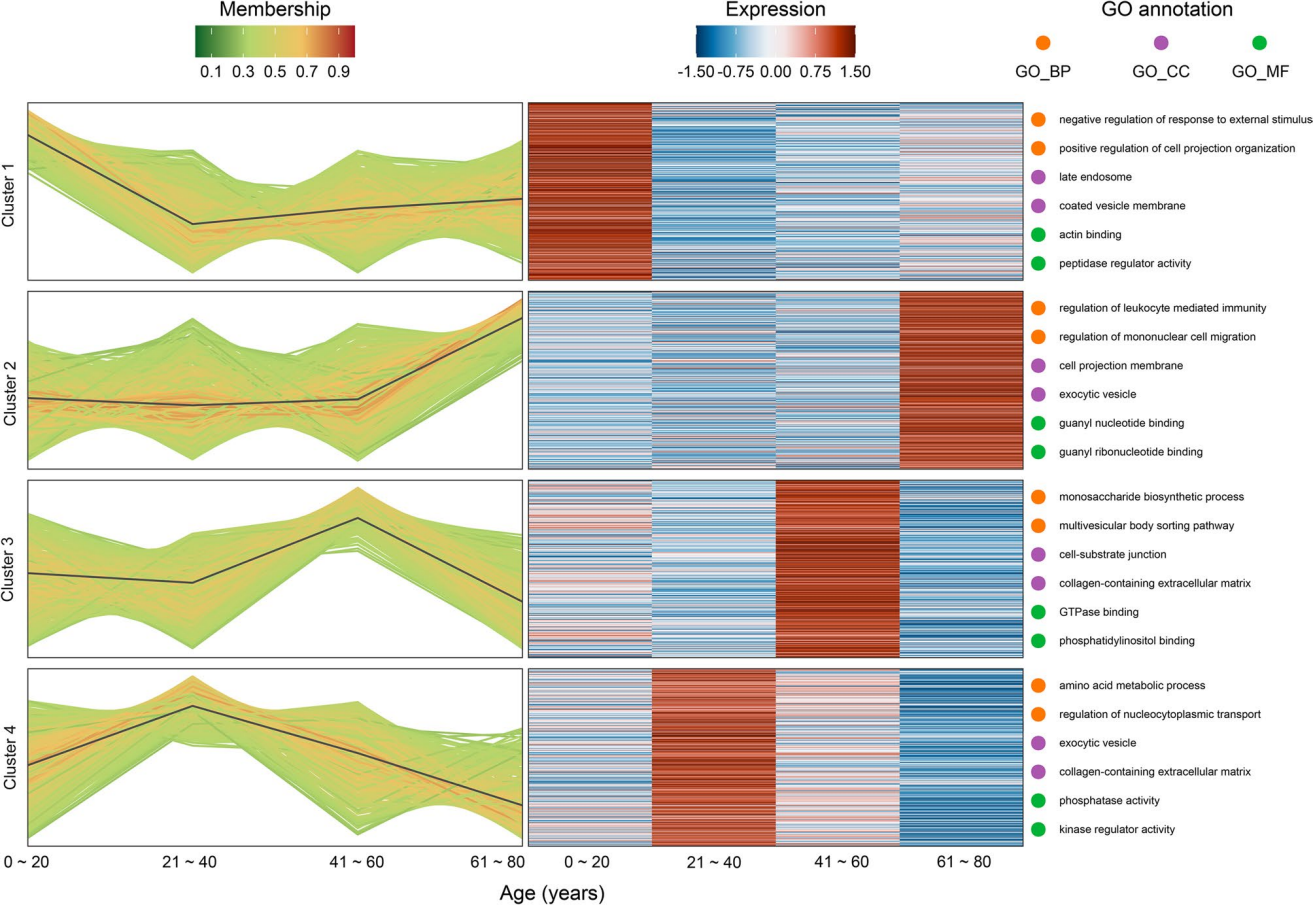
Mfuzz analysis reveals different expression patterns of proteins during aging. Line graph of four protein expression clusters (Cluster 1 to Cluster 4) is shown on the left, a protein expression heatmap is shown in the middle, and the top 2 GO enrichment analysis entries are shown on the right. Line graph: the X-axis represents age, Y-axis represents the relative protein expression, a line represents a protein, and the color of the line indicates the affiliation intensity in the cluster. Heatmap: the X-axis represents age, Y-axis represents different proteins, and heatmap color indicates the relative expression of the protein in the sample. Biological Process (BP) enrichment is in orange, Cellular Component (CC) enrichment is in purple, and Molecular Function (MF) enrichment is in green

We performed Mfuzz analyses to assess the expression levels and dynamics of 1,655 bloodstain proteins in four age groups (0–20, 21–40, 41–60, and 61–80 years) during the aging process. Results showed that all proteins were classified into four patterns according to the trend of change (Cluster 1 to Cluster 4, Fig. 2). Notably, Cluster 1 (including 400 proteins) displayed a decreasing trend, whereas Cluster 2 (including 458 proteins) exhibited an increasing trend. Cluster 3 (including 355 proteins) and Cluster 4 (including 442 proteins) displayed significant fluctuations in protein expression, with the highest protein abundance occurring between the ages of 41–60 years and 21–40 years, respectively.

Furthermore, GO analysis (including BP, MF, and CC categories) revealed that the proteins in each cluster exhibit distinct biological functions. For instance, the downregulated proteins (Cluster 1) were predominantly associated with negative regulation of response to external stimulus and positive regulation of cell projection organization, whereas the upregulated proteins (Cluster 2) were primarily enriched in the regulation of leukocyte-mediated immunity and mononuclear cell migration. The proteins in Cluster 3 are mainly involved in the monosaccharide biosynthetic process, and their MF is significantly enriched in GTPase binding and phosphatidylinositol binding, whereas Cluster 4 is closely related to the amino acid metabolic process and is characterized by phosphatase activity and kinase regulator activity as MFs.

### Age-associated proteomic signatures

**Fig. 3:**
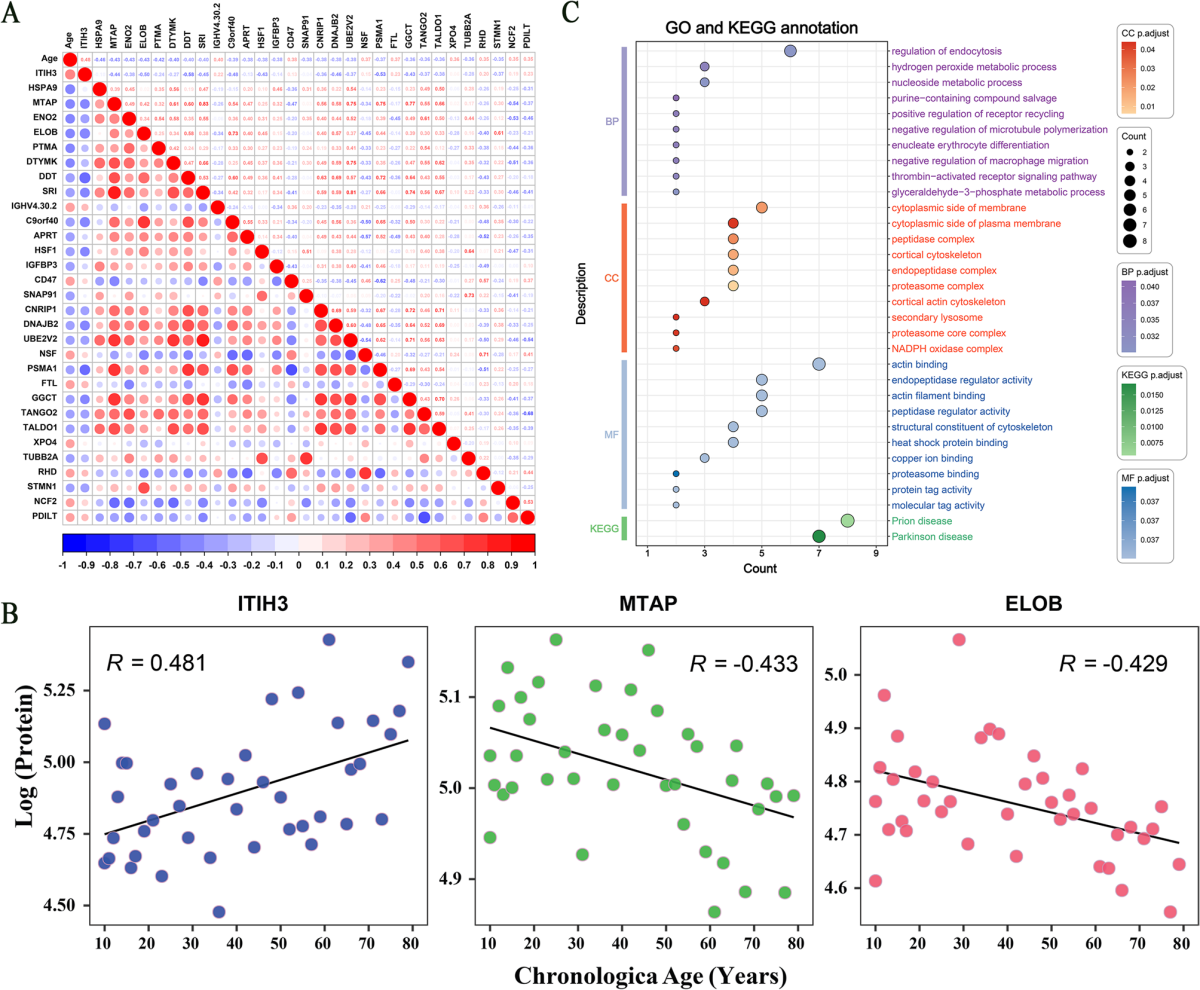
Correlation analysis of bloodstain proteins with age. **A** Correlation heat map of the age-associated proteins with |R| > 0.35 and *P* < 0.05. **B** Scatter plots showing the correlation of *ITIH3*, *MTAP*, and *ENO2* with age. **C** The top 10 GO and KEGG enriched all identified age-associated proteins (P. adjust < 0.05)

With criteria of *P* < 0.05 and |R| > 0.3, we identified the 71 proteins in bloodstains with the highest correlation with aging, including 26 positively correlated proteins and 45 negatively correlated proteins. Detailed information on these proteins can be found in Table S2. The correlation of bloodstain proteins with age is shown in Fig. 3. The correlation heat map of the proteins with |R| > 0.35 is shown in Fig. 3A. *ITIH3* showed the strongest association with age (*R* = 0.481). The remaining top 10 most significant proteins included *HSPA9* (*R* = − 0.459), *MTAP* (*R* = − 0.433), *ENO2* (*R* = − 0.431), *ELOB* (*R* = − 0.430), *PTMA* (*R* = − 0.419), *DTYMK* (*R* = − 0.405), *DDT* (*R* = 0.399), *SRI* (*R* = − 0.398), and *IGHV4-30-2* (*R* = − 0.397). Scatter plots showing the correlation of three proteins (*ITIH3*, *MTAP*, and *ENO2*) with age are shown in Fig. 3B.

We performed GO and KEGG enrichment analyses to evaluate the functional significance of all identified age-associated proteins. The top 10 enriched results in each category are shown in Fig. 3C and Table S3. GO analysis with P. adjust < 0.05 showed that the most enriched BP terms were: ‘regulation of endocytosis’, ‘hydrogen peroxide metabolic process’, and ‘nucleoside metabolic process’. The most enriched CC terms were: ‘cytoplasmic side of membrane’, ‘cytoplasmic side of plasma membrane’, and ‘peptidase complex’. The most enriched MF terms were: ‘actin binding’, ‘endopeptidase regulator activity’, and ‘actin filament binding’. Only two terms exist in the KEGG enrichment results (P. adjust < 0.05): ‘Prion disease’ and ‘Parkinson’s disease’, both of which are debilitating and age-related degenerative diseases.

### Construction of age-predictive models

**Fig. 4:**
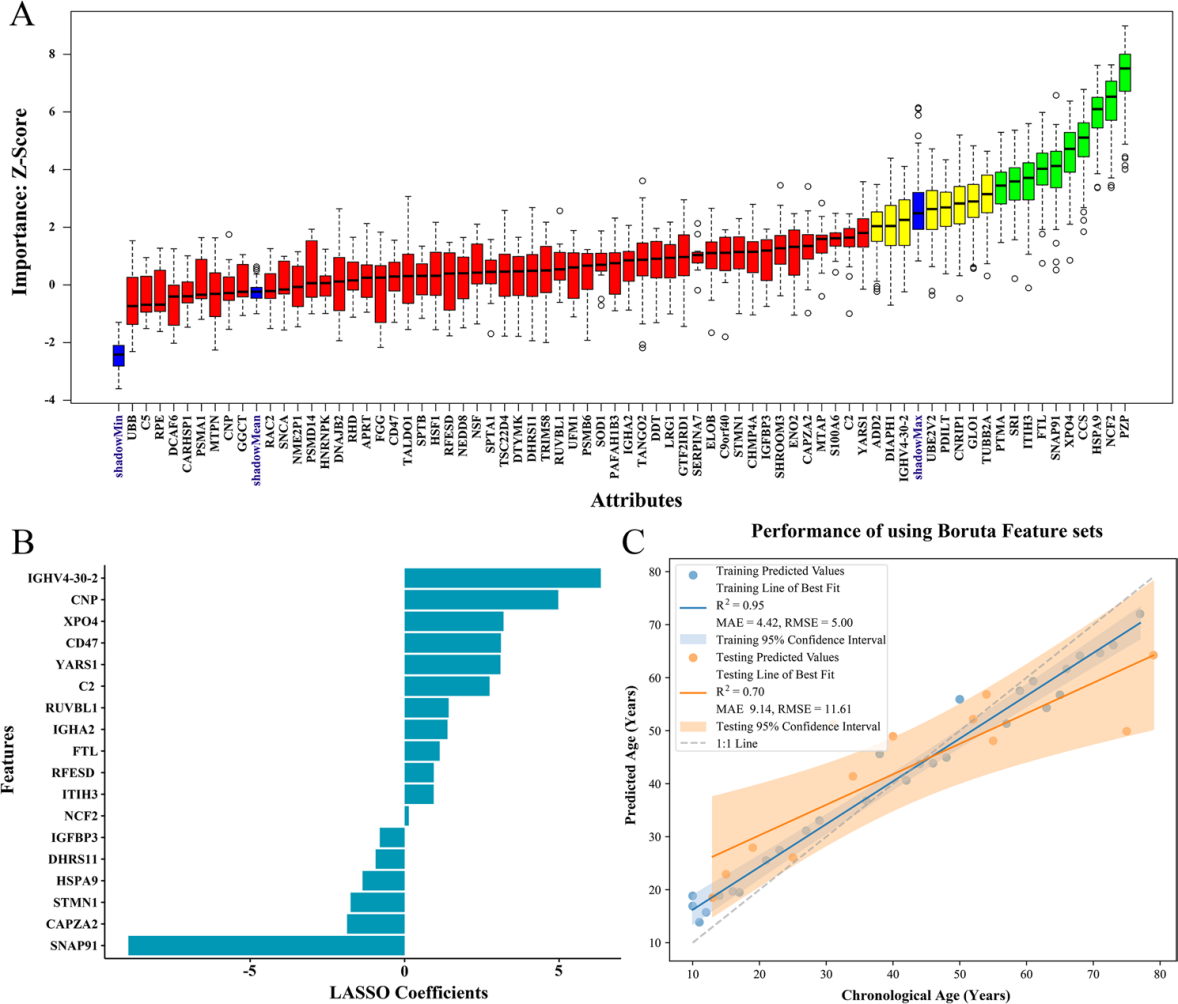
Results of model construction for feature screening. **A** Feature selection based on the Boruta algorithm. The horizontal axis is the gene name corresponding to each protein and the vertical axis is the Z-value of each protein. Green boxes represent significant variables, yellow boxes represent uncertain variables, and red boxes represent insignificant variables. **B** Feature selection based on the Lasso algorithm. The horizontal axis shows the coefficients of each protein, and the vertical axis shows the gene name corresponding to each protein. **C** Age prediction model constructed via RF machine learning using the 10 proteins selected by Boruta feature screening

The 71 bloodstain proteins from the correlation analysis were screened using two feature selection methods. The result of feature screening based on the Boruta algorithm is shown in Fig. 4A. In order of Z-scores, the 10 proteins most closely associated with age were *ITIH3*, *HSPA9*, *PTMA*, *SRI*, *SNAP91*, *FTL*, *XPO4*, *NCF2*, *PZP*, and *CCS*. The minimum λ value (1.290375) was selected, and 18 proteins with non-zero regression coefficients were identified using Lasso regression analysis (Fig. 4B). Together, six intersecting proteins were screened using these two methods: *ITIH3*, *HSPA9*, *SNAP91*, *FTL*, *XPO4*, and *NCF2*.

The RF age prediction models were further constructed for comparative analysis of different feature sets (Table 1). The model constructed using the 10 proteins selected by Boruta demonstrated optimal predictive performance, achieving R² = 0.95 and MAE = 4.42 years in the training set, and R² = 0.70 and MAE = 9.14 years in the testing set (Fig. 4C). For this optimal age prediction model, the best hyperparameters were found to be n_estimators = 300, max_features = ‘sqrt’, max_depth = 10, min_samples_split = 2, min_samples_leaf = 1. Furthermore, the model using the 18 proteins selected by Lasso regression achieved R² = 0.50 and MAE = 12.45 years in the testing set. Notably, simplified models constructed based on six intersecting proteins and two proteins most strongly correlated with age prediction performed comparably, yielding R² = 0.68 with MAE = 10.08 years and R² = 0.65 with MAE = 10.60 years in the testing set, respectively.

**Table 1:** Evaluation of the accuracy of age prediction in a training set and testing set using different feature sets based on the RF model

Feature sets	Training set			Testing set		
	*R* ^2^	MAE (Years)	RMSE (Years)	*R* ^2^	MAE (Years)	RMSE (Years)
|R| > 0.3 (71 proteins)	0.90	5.94	6.83	0.23	14.64	18.68
|R| > 0.35 (31 proteins)	0.92	5.37	6.15	0.22	14.58	18.75
|R | > 0.4 (7 proteins)	0.90	6.02	7.09	0.38	12.87	16.80
|R| > 0.45 (2 proteins)	0.93	4.92	5.78	0.65	10.60	12.58
Boruta (10 proteins)	**0.95**	**4.42**	**5.00**	**0.70**	**9.14**	**11.61**
Lasso (18 proteins)	0.96	5.25	5.95	0.50	12.45	14.98
Boruta/Lasso (6 proteins)	0.95	4.16	4.88	0.68	10.08	12.07

## Discussion

This study was the first systematic investigation of age-related proteomic changes in dried bloodstains using the Orbitrap Astral analyzer, a cutting-edge platform combining high sensitivity, resolution, and throughput. We described the proteome features of bloodstains that have not been previously described. Furthermore, we revealed the variations in bloodstain protein levels during aging and identified age-associated proteins and their related functions, suggesting that a proteomic predictor of age can be generated using a combination of these proteins.

We systematically analyzed the expression levels of 1,655 bloodstain proteins in four age groups (0–20, 21–40, 41–60, and 61–80 years) using Mfuzz cluster analysis, which revealed that they mainly show four clusters of protein trajectories that change with age. GO enrichment analyses showed that different proteins display unique changes during the aging process. For example, the levels of proteins involved in the regulation of leukocyte-mediated immunity and mononuclear cell migration remained constant until the 21–40 age group and then increased (Cluster 2), whereas the levels of proteins involved in amino acid-related BPs, metabolic processes, and the regulation of nucleocytoplasmic transport were the highest between the ages of 21 and 40 years and declined thereafter (Cluster 4). This revealed that the bloodstain proteome changes non-linearly over the human lifetime, which is in line with the results of previous studies [13]. This further reflects the complex changes in BPs.

We compared the 71 age-associated proteins identified in our study with those of Johnson’s review article on the proteomics of the aging clock [15]. *HSPA9*,* ENO2*, *CD47*, *DNAJB2*, *NSF*, *TALDO1*, *SNCA*, *SOD1*, *GLO1*, *HNRNPK*, *PAFAH1B3*, *C5*, *UFM1*, *IGHA2*, and *FGG*, which have been previously reported two or more times, were included in these 71 proteins. Of these, the plasma levels of *ENO2*, *CD47*, *DNAJB2*, *NSF*, *SNCA*, and *FGG* have been reported by two or more different studies to change significantly with age. Similarly, we detected these changes in bloodstains. Furthermore, *FGG* was reported up to six times, of which a progressive increase in *FGG* level with age has been reported four times, which is consistent with the results of the present study (*R* = 0.313). We also identified 56 proteins that were not mentioned in this review. For example, *PSMA1* has been found to play critical roles in protein homeostasis during aging in mice [22], whereas *TUBB2A* exhibited the highest downregulation across ages in almost all brain regions [23].

Seventy-one age-related proteins were enriched using GO and KEGG analyses. The most significant BP term was the regulation of endocytosis, which is involved in the transport of a wide range of transmembrane receptors and their cargoes from the cell surface to the cell interior. Studies have shown that cellular senescence is associated with both cytochalasin-mediated and cisternae-mediated endocytosis [24]. Second was hydrogen peroxide metabolism, which is involved in most of the redox metabolic reactions and processes of the cell [25]. Hydrogen peroxide induces DNA damage in the tumor microenvironment, leading to premature aging [26]. Moreover, an increased level of mitochondrial hydrogen peroxide is associated with Alzheimer’s disease and brain aging [27]. KEGG enrichment further connected these 71 proteins to age-related neurodegenerative diseases such as Prion and Parkinson’s disease, potentially indicating an imbalance in protein homeostasis [28].

Among the six intersecting proteins (*ITIH3*, *HSPA9*, *SNAP91*, *FTL*, *XPO4*, and *NCF2*), *ITIH3* (*R* = 0.481) and *HSPA9* (*R* = – 0.459) were the age-associated proteins most strongly correlated with age in this study. *ITIH3* belongs to the *ITI* family, which is associated with inflammation and carcinogenesis [29]. *ITIH3* levels are increased in the plasma of elderly individuals [30], and we also observed a similar trend in bloodstain samples, suggesting its potential as a stable biomarker of aging. *HSPA9* (also called *GRP75* and Mortalin) is a member of the 70-kDa *HSP70* family that modulates mitochondrial dynamics in neurons [31], where mitochondrial functions were reported to cause accelerated aging [32]. *HSPA9* is also involved in sensing of oxidative stress and activation of antioxidant pathways [33], both of which are closely linked to aging. *SNAP91*, also known as clathrin assembly protein 180 (*AP180*), is enriched in the presynaptic terminal of neurons [34] and plays an essential role in synaptic neurotransmission. *HSPA9* and *SNAP91* are associated with neurodegenerative diseases, including Parkinson’s [35, 36] and Alzheimer’s diseases [37, 38], emphasizing their roles in physiological aging and age-related disease progression. *FTL*, a subunit of ferritin, is essential for maintaining iron homeostasis in organisms. Recent studies reveal that *FTL1* in mouse brains accumulates progressively during aging and is associated with cognitive decline [39]. *XPO4* is a member of the importin β family that mediates the nuclear-cytoplasmic transport of protein cargoes [40]. The direct association between *XPO4* and aging remains inconclusive at present. We speculate that *XPO4* may participate in the transforming growth factor beta 1 (*TGF-β1*)/Smad3 signaling pathway, thereby influencing aging [41, 42]. *NCF2* (also known as *p67*^*phox*^) is a cytoplasmic component of a multiprotein complex *NADPH* oxidase, which is involved in the production of reactive oxygen species (ROS) in phagocytic cells [43]. ROS plays a key role in tissue damage and aging [44], with *NADPH* oxidase being a key factor in ROS accumulation during these processes [45]. Moreover, *NCF2* expression levels increase in healthy elderly individuals [46]. In summary, all these results may serve as references for further exploration on the function of these six intersecting proteins.

Although DNA methylation is widely recognized as the ideal biomarker for age prediction, its application to bloodstains, the most common biological evidence at crime scenes, has not been studied extensively. Current research has focused on fresh whole-blood samples with a MAE of 3–10 years [47]. By contrast, studies on bloodstains have shown inconsistent performance. Huang et al. reported 7–8 years of MAE using a methylation-based model [48], whereas Yang et al. achieved higher accuracy (MAE < 3 years) [47]. Furthermore, the use of DNA methylation as a marker has several limitations. For example, the risk of DNA breakage or degradation can be influenced by various factors such as lifestyle [6, 7], which may decrease the accuracy of detection results. These diverse findings highlight the need to further explore the age prediction performance for stained biological samples. To address this challenge, we developed a proteomic approach for bloodstain age prediction. Utilizing R as a screening criterion, we stratified the 71 candidate proteins into four subgroups (|R| > 0.3, > 0.35, > 0.4, and > 0.45) and developed corresponding prediction models. We found that reducing the number of proteins did not decrease the accuracy of age prediction. Instead, the high prediction accuracy (R^2^ = 0.65 and MAE = 10.60 years in the testing set) was obtained using the only two proteins with the highest correlation (*ITIH3* and *HSPA9*). The Boruta and Lasso algorithms have become the most widely used methods in feature selection. By contrast, the age prediction model based on the 10 proteins screened using Boruta performed the best and achieved a MAE of 9.14 years for the testing set.

Despite a disparity between the performance of these bloodstain proteomic models and established epigenetic methods for age prediction, unique advantages exist. On the one hand, protein-based modeling overcomes the limitations of bisulfite conversion and provides supplementary information when DNA detection is unavailable. On the other hand, to our knowledge, this is the first study addressing the potential of bloodstain proteins in age prediction, demonstrating the feasibility of this direction and laying the foundation for future development.

Previous studies have demonstrated that bloodstain proteins stored on FTA cards exhibit excellent long-term stability. For example, Björkesten et al. reported that the detection of certain proteins remained significantly unaffected by storage over thirty years [17]. Sun et al., Eshghi et al., and Chambers et al., respectively, confirmed that the majority of proteins were stable in dried bloodstain samples after 6 months [49], 57 days [19], and 154 days [18] at controlled temperatures. In this study, the high protein quality and detection coverage of bloodstain samples stored for 4 years under standard laboratory conditions were confirmed through Bradford assay, SDS-PAGE, and mass spectrometry-based proteomics analysis. This further demonstrates the excellent temporal stability of bloodstain proteomes under controlled environmental conditions. These results support the age-related changes observed in this study, which primarily reflect biological aging processes rather than storage-related interference. However, this stability may not extend to harsh and uncontrolled environments (e.g., UV exposure, temperature and humidity fluctuations, and microbial activity) [50]. Furthermore, dried bloodstains are usually deposited on various carriers at actual crime scenes. Although some studies have achieved recovery of bloodstain proteins from complex substrates for downstream mass spectrometry analysis [51–54], protein recovery efficiency may be influenced by the type of carrier material.

In summary, we will expand our research in the following four aspects in the future. Firstly, expanding the sample size to identify more key aging proteins (such as *GDF15*), as the smaller sample size lacks overall representativeness [14]. Secondly, in practical applications, the gender of donors for biological evidence collected at crime scenes is often uncertain (possibly male or female), so the final age prediction model must be universal for both genders. Therefore, subsequent research should expand the cohort to achieve gender balance and compare combined models with gender-stratified models, ensuring the model demonstrates universal and equitable performance across both male and female populations. Thirdly, the consistency of results obtained from two independent feature selection methods (Lasso regression and Boruta algorithm) in this study, along with the identification of age-related proteins consistent with previous reports, confirms the reliability of our findings. However, subsequent work should still validate these biomarkers through traditional biochemical or targeted proteomics methods in larger cohort studies to enhance their potential for forensic application. Finally, considering the complexity of crime scene environments, future studies must focus on the protein recovery and the stability of the age-specific protein biomarkers identified under broader forensic-related environmental conditions and across different storage durations. We believe that conducting further experimental studies will bring intriguing results and provide a broad mind for forensic proteomics-based age estimation.

## Conclusion

In this pilot study, we identified 1,655 bloodstain proteins in bloodstain samples using the proteomic approach. These proteins showed four different patterns of change during the aging process. Seventy-one bloodstain proteins (including 26 positively and 45 negatively correlated proteins) were identified as age-related, and functional enrichment further connected these proteins to senescence-related pathways. We then constructed age prediction models with applicability in this pilot study, and the Boruta-based model demonstrated the highest predictive accuracy, achieving an R² of 0.70 and a MAE of 9.14 years for the testing set. We also present the shortcomings of the study and future perspectives. In summary, the results of this pilot study corroborate those of proteomic aging studies in bloodstains and establish bloodstain proteomics as a novel tool for forensic age estimation.

## Supplementary Information

Supplementary Material 1

Supplementary Material 2

## Data Availability

The mass spectrometry proteomics data have been deposited to the ProteomeXchange Consortium via the iProX partner repository with the dataset identifier PXD066907.

## Abbreviations

ACN: Acetonitrile
BP: Biological Process
CC: Cellular Component
COGs: Clusters of Orthologous Groups of proteins
DIA: Data-independent acquisition
FA: Formic acid
FDR: False discovery rate
FTA: Flinders Technology Associates
GO: Gene Ontology
IPR: InterPro
KEGG: Kyoto Encyclopedia of Genes and Genomes
Lasso: Least Absolute Shrinkage and Selection Operator
LC: Liquid Chromatography
MS: Mass Spectrometry
MAE: Mean absolute Error
MF: Molecular Function
PSMs: Peptide spectrum matches
ROS: Reactive oxygen species
RF: Random Forest
RMSE: Root Mean Square Error
SDS-PAGE: Sodium dodecyl sulfate polyacrylamide gel electrophoresis
TEAB: Triethylammonium bicarbonate
