# Supplementary material for: Proteomic signature of aging in bloodstain samples: a preliminary study
Source: BMC Genomics. 2025 Oct 29;26:970. doi: 10.1186/s12864-025-12164-x (PMC12573856; doi:10.1186/s12864-025-12164-x)
Supplement: Supplementary file 1 — Supplementary Material 1 [file 12864_2025_12164_MOESM1_ESM.docx]

**Supplementary Figures**

**Fig. S1**


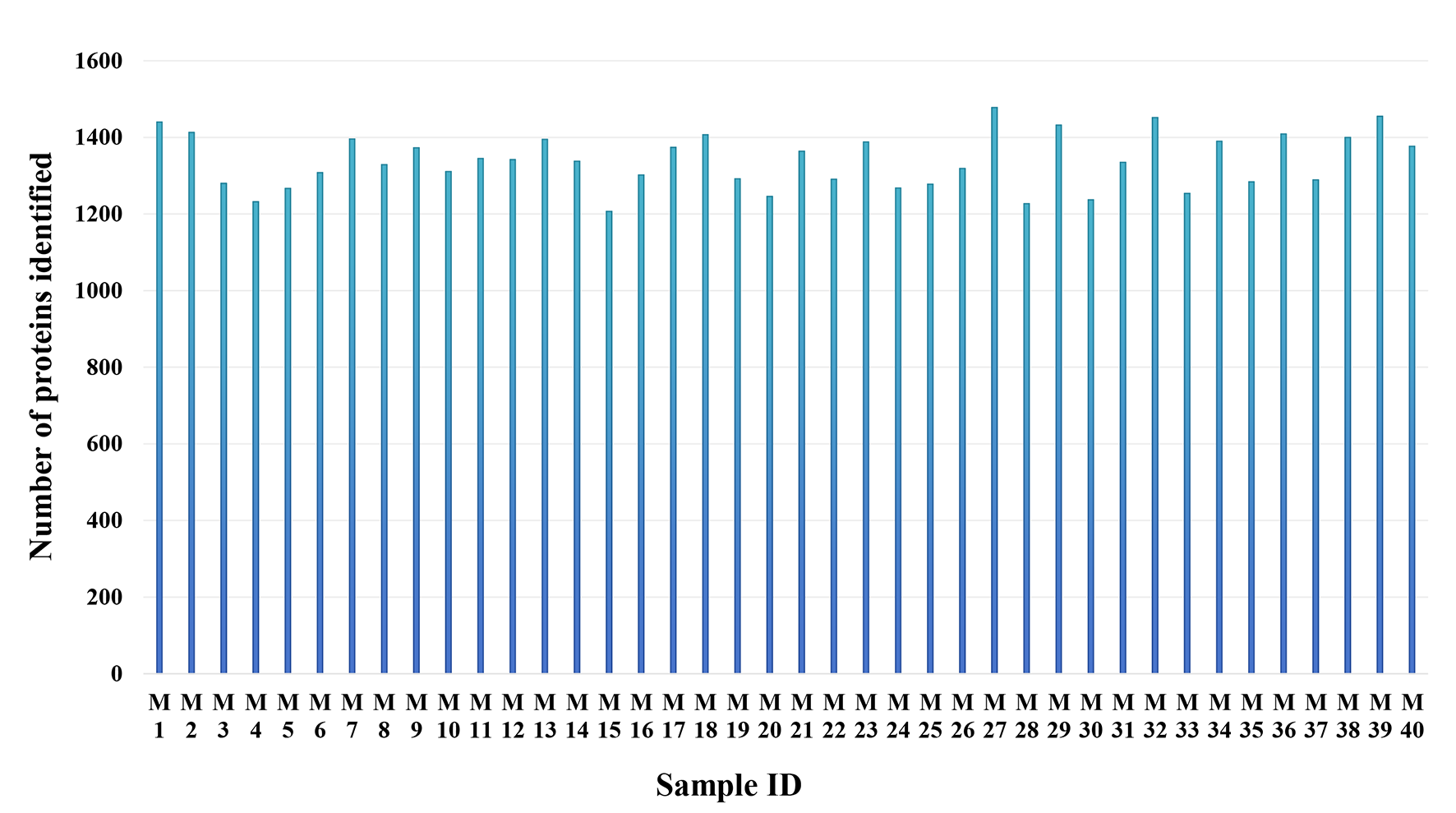


**Fig. S1.** The number of proteins detected in each sample in this study.

**Fig. S2**

**
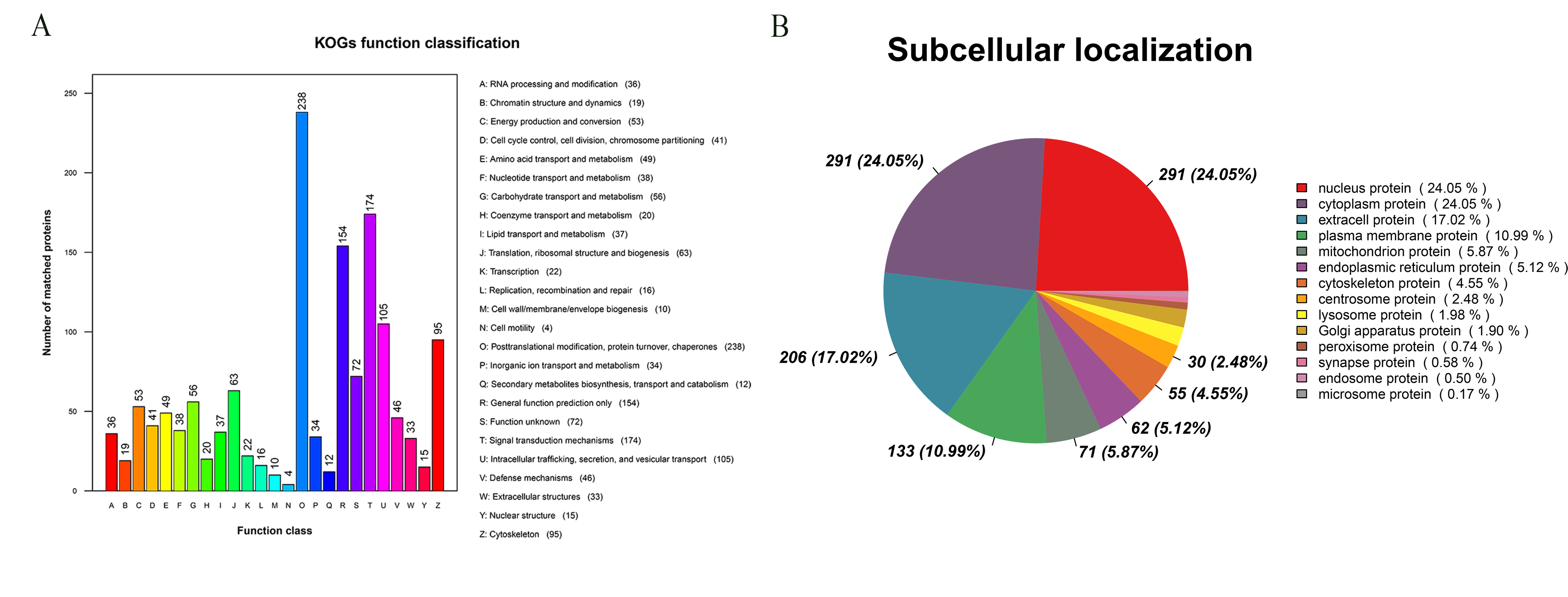
**

**Fig. S2.** (A) The top 20 KOG function class. (B) Subcellular localization of all identified bloodstains proteins.
