# Supplementary material for: Proteomic signature of aging in bloodstain samples: a preliminary study
Source: BMC Genomics. 2025 Oct 29;26:970. doi: 10.1186/s12864-025-12164-x (PMC12573856; doi:10.1186/s12864-025-12164-x)
Supplement: Supplementary file 2 — Supplementary Material 2 [file 12864_2025_12164_MOESM2_ESM.docx]

**Supplementary Tables**

**Table S1.** Liquid chromatography gradient elution program for proteomic analysis.

| **Time** | **Flow Rate (µL/min)** | **Mobile Phase A (%)** | **Mobile Phase B (%)** |
| --- | --- | --- | --- |
| 0 | 2.5 | 96 | 4 |
| 0.2 | 2 | 96 | 4 |
| 0.3 | 1.5 | 92 | 8 |
| 7.5 | 1.5 | 77.5 | 22.5 |
| 12.2 | 1.5 | 65 | 35 |
| 12.6 | 2.5 | 45 | 55 |
| 12.6 | Column Wash | | |
| 13 | 2.5 | 1 | 99 |
| 13.7 | 2.5 | 1 | 99 |
| 13.7 | Stop Run | | |

**Table S2.** Age-associated proteins identified in bloodstains (|R| > 0.3 and P < 0.05).

| **Uniprot** | **Gene** | **R** | **R.squared** | **p.value** |
| --- | --- | --- | --- | --- |
| Q06033 | ITIH3 | 0.481445789 | 0.231790048 | 0.001659781 |
| P38646 | HSPA9 | -0.458510695 | 0.210232057 | 0.002924939 |
| Q13126 | MTAP | -0.432732271 | 0.187257219 | 0.005289355 |
| P09104 | ENO2 | -0.43051825 | 0.185345964 | 0.005553923 |
| Q15370 | ELOB | -0.429016133 | 0.184054843 | 0.005739877 |
| P06454 | PTMA | -0.418544114 | 0.175179176 | 0.007192414 |
| P23919 | DTYMK | -0.404547548 | 0.163658718 | 0.00962039 |
| P30046 | DDT | -0.398917875 | 0.159135471 | 0.010778322 |
| P30626 | SRI | -0.398158175 | 0.158529932 | 0.010943329 |
| A0A087WSY4 | IGHV4-30-2 | 0.396772541 | 0.157428449 | 0.011249841 |
| Q8IXQ3 | C9orf40 | -0.393162145 | 0.154576473 | 0.012083051 |
| P07741 | APRT | -0.384895084 | 0.148144226 | 0.014190557 |
| Q00613 | HSF1 | -0.384476017 | 0.147821808 | 0.014305195 |
| P17936 | IGFBP3 | -0.383335288 | 0.146945943 | 0.014621225 |
| Q08722 | CD47 | 0.381486451 | 0.145531912 | 0.01514597 |
| O60641 | SNAP91 | -0.380828589 | 0.145030414 | 0.015336486 |
| Q96F85 | CNRIP1 | -0.379512934 | 0.144030067 | 0.015723575 |
| P25686 | DNAJB2 | -0.378813168 | 0.143499416 | 0.015932797 |
| Q15819 | UBE2V2 | -0.37730346 | 0.142357901 | 0.016392193 |
| P46459 | NSF | 0.371757203 | 0.138203418 | 0.018176918 |
| P25786 | PSMA1 | -0.368957743 | 0.136129816 | 0.019138039 |
| P02792 | FTL | 0.367695314 | 0.135199844 | 0.019585206 |
| O75223 | GGCT | -0.363085312 | 0.131830944 | 0.021293203 |
| Q6ICL3 | TANGO2 | -0.36269242 | 0.131545791 | 0.021444354 |
| P37837 | TALDO1 | -0.36210837 | 0.131122471 | 0.021670702 |
| Q9C0E2 | XPO4 | 0.360356834 | 0.129857048 | 0.022361506 |
| Q13885 | TUBB2A | -0.355776201 | 0.126576705 | 0.024255429 |
| Q02161 | RHD | 0.354135161 | 0.125411712 | 0.024965605 |
| P16949 | STMN1 | -0.353525488 | 0.124980271 | 0.02523381 |
| P19878 | NCF2 | 0.353399702 | 0.124891349 | 0.025289443 |
| Q8N807 | PDILT | 0.353066673 | 0.124656076 | 0.025437225 |
| O00487 | PSMD14 | -0.349271874 | 0.121990842 | 0.027172408 |
| P20742 | PZP | 0.34706785 | 0.120456092 | 0.028224437 |
| P09543 | CNP | 0.346619038 | 0.120144757 | 0.02844274 |
| P15153 | RAC2 | 0.345453951 | 0.119338432 | 0.029015962 |
| Q8TF72 | SHROOM3 | -0.342194107 | 0.117096807 | 0.030670699 |
| P06703 | S100A6 | -0.341869006 | 0.116874417 | 0.030839904 |
| Q6UWP2 | DHRS11 | -0.339616743 | 0.115339532 | 0.032033378 |
| P28072 | PSMB6 | -0.339585168 | 0.115318086 | 0.032050376 |
| Q9BY43 | CHMP4A | -0.338514908 | 0.114592343 | 0.032630932 |
| P06681 | C2 | 0.338226382 | 0.114397085 | 0.032788911 |
| O60361 | NME2P1 | -0.337410015 | 0.113845518 | 0.033239313 |
| P37840 | SNCA | -0.336911089 | 0.113509082 | 0.033517071 |
| P02549 | SPTA1 | 0.333474057 | 0.111204947 | 0.035482707 |
| Q8NG06 | TRIM58 | 0.333072848 | 0.110937522 | 0.035718185 |
| P35612 | ADD2 | 0.332263921 | 0.110399313 | 0.036196853 |
| P0CG47 | UBB | -0.331982955 | 0.110212682 | 0.036364332 |
| Q9UHL9 | GTF2IRD1 | 0.33195767 | 0.110195895 | 0.036379435 |
| O60610 | DIAPH1 | 0.330712528 | 0.109370776 | 0.037129544 |
| Q96AT9 | RPE | -0.33022437 | 0.109048134 | 0.037427051 |
| P00441 | SOD1 | -0.328747445 | 0.108074883 | 0.038339036 |
| Q58WW2 | DCAF6 | -0.3280393 | 0.107609782 | 0.038782695 |
| P58546 | MTPN | -0.327039447 | 0.1069548 | 0.039416229 |
| O14618 | CCS | -0.326170758 | 0.106387363 | 0.039973476 |
| Q8TAC1 | RFESD | -0.326103139 | 0.106343257 | 0.04001712 |
| Q04760 | GLO1 | -0.32586868 | 0.106190396 | 0.04016875 |
| P47755 | CAPZA2 | -0.322051386 | 0.103717095 | 0.042703953 |
| P61978 | HNRNPK | -0.321643444 | 0.103454505 | 0.042982392 |
| P02750 | LRG1 | 0.321607163 | 0.103431167 | 0.043007227 |
| Q9Y2V2 | CARHSP1 | -0.320643095 | 0.102811994 | 0.043671415 |
| Q9Y265 | RUVBL1 | 0.320423305 | 0.102671095 | 0.043823995 |
| P11277 | SPTB | 0.319398809 | 0.102015599 | 0.044540922 |
| Q15102 | PAFAH1B3 | -0.318804389 | 0.101636239 | 0.044961221 |
| P05543 | SERPINA7 | 0.318070312 | 0.101168723 | 0.045484692 |
| P54577 | YARS1 | 0.317536662 | 0.100829532 | 0.045868324 |
| Q9Y3Q8 | TSC22D4 | -0.315936933 | 0.099816146 | 0.04703404 |
| P01031 | C5 | 0.314436415 | 0.098870259 | 0.048149088 |
| P01877 | IGHA2 | 0.314034891 | 0.098617913 | 0.048451051 |
| P02679 | FGG | 0.312863716 | 0.097883705 | 0.049340551 |
| Q15843 | NEDD8 | -0.312714974 | 0.097790655 | 0.049454455 |
| P61960 | UFM1 | -0.312337571 | 0.097554758 | 0.049744412 |

**Table S3.** GO and KEGG enrichment analysis of the 71 age-associated proteins.

| **Oategory** | **ID** | **Description** | **GeneRatio** | **BgRatio** | **Rich**  **Factor** | **Fold**  **Enrichment** | **zScore** | **P-value** | **p.adjust** | **q-value** | **geneID** | **Count** |
| --- | --- | --- | --- | --- | --- | --- | --- | --- | --- | --- | --- | --- |
| BP | GO:0030100 | regulation of endocytosis | 6/67 | 307/18986 | 0.019543974 | 5.538237153 | 4.770601903 | 0.00074229 | 0.030092225 | 0.025708922 | CD47/SNAP91/C2/SNCA/SOD1/HNRNPK | 6 |
| BP | GO:0042743 | hydrogen peroxide metabolic process | 3/67 | 55/18986 | 0.054545455 | 15.45671642 | 6.38936712 | 0.000966482 | 0.035115508 | 0.030000501 | RAC2/SNCA/SOD1 | 3 |
| BP | GO:0009116 | nucleoside metabolic process | 3/67 | 50/18986 | 0.06 | 17.00238806 | 6.742474166 | 0.000731192 | 0.030092225 | 0.025708922 | MTAP/DTYMK/APRT | 3 |
| BP | GO:0043101 | purine-containing compound salvage | 2/67 | 15/18986 | 0.133333333 | 37.78308458 | 8.480906529 | 0.001250492 | 0.040891104 | 0.034934811 | MTAP/APRT | 2 |
| BP | GO:0001921 | positive regulation of receptor recycling | 2/67 | 14/18986 | 0.142857143 | 40.48187633 | 8.794251007 | 0.001086232 | 0.036244688 | 0.030965203 | NSF/SNCA | 2 |
| BP | GO:0031115 | negative regulation of microtubule polymerization | 2/67 | 14/18986 | 0.142857143 | 40.48187633 | 8.794251007 | 0.001086232 | 0.036244688 | 0.030965203 | STMN1/SNCA | 2 |
| BP | GO:0043353 | enucleate erythrocyte differentiation | 2/67 | 14/18986 | 0.142857143 | 40.48187633 | 8.794251007 | 0.001086232 | 0.036244688 | 0.030965203 | RAC2/TRIM58 | 2 |
| BP | GO:1905522 | negative regulation of macrophage migration | 2/67 | 14/18986 | 0.142857143 | 40.48187633 | 8.794251007 | 0.001086232 | 0.036244688 | 0.030965203 | DDT/C5 | 2 |
| BP | GO:0070493 | thrombin-activated receptor signaling pathway | 2/67 | 13/18986 | 0.153846154 | 43.59586682 | 9.142495322 | 0.000933181 | 0.034676139 | 0.029625132 | STMN1/SNCA | 2 |
| BP | GO:0019682 | glyceraldehyde-3-phosphate metabolic process | 2/67 | 12/18986 | 0.166666667 | 47.22885572 | 9.53274404 | 0.000791416 | 0.030092225 | 0.025708922 | TALDO1/RPE | 2 |
| CC | GO:0098562 | cytoplasmic side of membrane | 5/67 | 210/19960 | 0.023809524 | 7.093105899 | 5.151354916 | 0.000699135 | 0.021273693 | 0.017977769 | DNAJB2/S100A6/CHMP4A/SPTA1/SPTB | 5 |
| CC | GO:0009898 | cytoplasmic side of plasma membrane | 4/67 | 163/19960 | 0.024539877 | 7.310685835 | 4.694910525 | 0.002202711 | 0.042652486 | 0.036044354 | S100A6/CHMP4A/SPTA1/SPTB | 4 |
| CC | GO:1905368 | peptidase complex | 4/67 | 129/19960 | 0.031007752 | 9.237533264 | 5.447238345 | 0.000931657 | 0.024805358 | 0.020962274 | DNAJB2/PSMA1/PSMD14/PSMB6 | 4 |
| CC | GO:0030863 | cortical cytoskeleton | 4/67 | 109/19960 | 0.036697248 | 10.9324935 | 6.034440267 | 0.000495011 | 0.017572878 | 0.01485032 | SHROOM3/SPTA1/CAPZA2/SPTB | 4 |
| CC | GO:1905369 | endopeptidase complex | 4/67 | 98/19960 | 0.040816327 | 12.15961011 | 6.426984018 | 0.00033042 | 0.014075908 | 0.011895134 | DNAJB2/PSMA1/PSMD14/PSMB6 | 4 |
| CC | GO:0000502 | proteasome complex | 4/67 | 64/19960 | 0.0625 | 18.61940299 | 8.193226079 | 6.33E-05 | 0.006269079 | 0.005297813 | DNAJB2/PSMA1/PSMD14/PSMB6 | 4 |
| CC | GO:0030864 | cortical actin cytoskeleton | 3/67 | 80/19960 | 0.0375 | 11.17164179 | 5.290351278 | 0.002469604 | 0.043835472 | 0.037044061 | SHROOM3/SPTA1/SPTB | 3 |
| CC | GO:0005767 | secondary lysosome | 2/67 | 23/19960 | 0.086956522 | 25.90525633 | 6.935562322 | 0.002683263 | 0.043964225 | 0.037152866 | FTL/NCF2 | 2 |
| CC | GO:0005839 | proteasome core complex | 2/67 | 20/19960 | 0.1 | 29.79104478 | 7.475951838 | 0.002028222 | 0.042652486 | 0.036044354 | PSMA1/PSMB6 | 2 |
| CC | GO:0043020 | NADPH oxidase complex | 2/67 | 18/19960 | 0.111111111 | 33.10116086 | 7.907319437 | 0.00164034 | 0.038821382 | 0.032806802 | NCF2/RAC2 | 2 |
| MF | GO:0003779 | actin binding | 7/68 | 441/18737 | 0.015873016 | 4.373716153 | 4.326952492 | 0.001073499 | 0.036805221 | 0.028339302 | SHROOM3/SNCA/SPTA1/ADD2/DIAPH1/CAPZA2/SPTB | 7 |
| MF | GO:0061135 | endopeptidase regulator activity | 5/68 | 189/18737 | 0.026455026 | 7.289526922 | 5.244845582 | 0.000617202 | 0.036805221 | 0.028339302 | ITIH3/PSMD14/PZP/SERPINA7/C5 | 5 |
| MF | GO:0051015 | actin filament binding | 5/68 | 211/18737 | 0.023696682 | 6.529481461 | 4.87491639 | 0.001012417 | 0.036805221 | 0.028339302 | SHROOM3/SPTA1/ADD2/CAPZA2/SPTB | 5 |
| MF | GO:0061134 | peptidase regulator activity | 5/68 | 235/18737 | 0.021276596 | 5.862640801 | 4.527194465 | 0.001630902 | 0.036805221 | 0.028339302 | ITIH3/PSMD14/PZP/SERPINA7/C5 | 5 |
| MF | GO:0005200 | structural constituent of cytoskeleton | 4/68 | 113/18737 | 0.03539823 | 9.753774076 | 5.632879249 | 0.00075881 | 0.036805221 | 0.028339302 | TUBB2A/SPTA1/ADD2/SPTB | 4 |
| MF | GO:0031072 | heat shock protein binding | 4/68 | 131/18737 | 0.030534351 | 8.413560844 | 5.138878659 | 0.00131501 | 0.036805221 | 0.028339302 | HSPA9/HSF1/DNAJB2/SNCA | 4 |
| MF | GO:0005507 | copper ion binding | 3/68 | 63/18737 | 0.047619048 | 13.12114846 | 5.816044695 | 0.001553746 | 0.036805221 | 0.028339302 | SNCA/SOD1/CCS | 3 |
| MF | GO:0070628 | proteasome binding | 2/68 | 18/18737 | 0.111111111 | 30.61601307 | 7.586717115 | 0.001912454 | 0.037553639 | 0.02891557 | DNAJB2/PSMD14 | 2 |
| MF | GO:0031386 | protein tag activity | 2/68 | 14/18737 | 0.142857143 | 39.36344538 | 8.666151366 | 0.001148184 | 0.036805221 | 0.028339302 | UBB/NEDD8 | 2 |
| MF | GO:0141047 | molecular tag activity | 2/68 | 14/18737 | 0.142857143 | 39.36344538 | 8.666151366 | 0.001148184 | 0.036805221 | 0.028339302 | UBB/NEDD8 | 2 |
| KEGG | hsa05020 | Prion disease | 8/42 | 278/8536 | 0.028776978 | 5.84857828 | 5.779225625 | 5.15E-05 | 0.00551351 | 0.004935852 | PSMA1/TUBB2A/NCF2/PSMD14/RAC2/PSMB6/SOD1/C5 | 8 |
| KEGG | hsa05012 | Parkinson disease | 7/42 | 271/8536 | 0.025830258 | 5.249692497 | 4.999090537 | 0.000312912 | 0.01674078 | 0.014986827 | PSMA1/TUBB2A/PSMD14/RAC2/SNCA/UBB/SOD1 | 7 |
